# Application of a risk-management framework for integration of stromal tumor-infiltrating lymphocytes in clinical trials

**DOI:** 10.1038/s41523-020-0155-1

**Published:** 2020-05-12

**Authors:** Jan Hudeček, Leonie Voorwerk, Maartje van Seijen, Iris Nederlof, Michiel de Maaker, Jose van den Berg, Koen K. van de Vijver, Karolina Sikorska, Sylvia Adams, Sandra Demaria, Giuseppe Viale, Torsten O. Nielsen, Sunil S. Badve, Stefan Michiels, William Fraser Symmans, Christos Sotiriou, David L. Rimm, Stephen M. Hewitt, Carsten Denkert, Sibylle Loibl, Sherene Loi, John M. S. Bartlett, Giancarlo Pruneri, Deborah A. Dillon, Maggie C. U. Cheang, Andrew Tutt, Jacqueline A. Hall, Zuzana Kos, Roberto Salgado, Marleen Kok, Hugo M. Horlings, International Immuno-Oncology Biomarker Working Group, Aini Hyytiäinen, Akira I. Hida, Alastair Thompson, Alex Lefevre, Alexander J. Lazar, Allen Gown, Amy Lo, Anna Sapino, Anant Madabhushi, Andre Moreira, Andrea Richardson, Andrea Vingiani, Andrew H. Beck, Andrew M. Bellizzi, Angel Guerrero, Anita Grigoriadis, Anna Ehinger, Ana Garrido-Castro, Anne Vincent-Salomon, Anne-Vibeke Laenkholm, Ashish Sharma, Ashley Cimino-Mathews, Ashok Srinivasan, Balazs Acs, Baljit Singh, Benjamin Calhoun, Benjamin Haibe-Kans, Benjamin Solomon, Bibhusal Thapa, Brad H. Nelson, Brandon D. Gallas, Carlos Castaneda, Carmen Ballesteros-Merino, Carmen Criscitiello, Carolien Boeckx, Cecile Colpaert, Cecily Quinn, Chakra S. Chennubhotla, Charles Swanton, Cinzia Solinas, Crispin Hiley, Damien Drubay, Daniel Bethmann, David A. Moore, Denis Larsimont, Dhanusha Sabanathan, Dieter Peeters, Dimitrios Zardavas, Doris Höflmayer, Douglas B. Johnson, E. Aubrey Thompson, Edi Brogi, Edith Perez, Ehab A. ElGabry, Elisabeth Specht Stovgaard, Elizabeth F. Blackley, Elvire Roblin, Emily Reisenbichler, Enrique Bellolio, Eva Balslev, Ewa Chmielik, Fabien Gaire, Fabrice Andre, Fang-I Lu, Farid Azmoudeh-Ardalan, Federico Rojo, Tina Gruosso, Francesco Ciompi, Franklin Peale, Fred R. Hirsch, Frederick Klauschen, Frédérique Penault-Llorca, Gabriela Acosta Haab, Gelareh Farshid, Gert van den Eynden, Giuseppe Curigliano, Giuseppe Floris, Glenn Broeckx, Gonzalez-Ericsson, Harmut Koeppen, Harry R. Haynes, Heather McArthur, Heikki Joensuu, Helena Olofsson, Huang-Chun Lien, I-Chun Chen, Ian Cree, Isabel Frahm, Iva Brcic, Jack Chan, James Ziai, Jane Brock, Jelle Wesseling, Jennifer Giltnane, Jennifer K. Kerner, Jeppe Thagaard, Jeremy P. Braybrooke, Jeroen A. W. M. van der Laak, Jerome Lemonnier, Jiping Zha, Joana Ribeiro, Jochen K. Lennerz, Jodi M. Carter, Joel Saltz, Johan Hartman, Johannes Hainfellner, John Le Quesne, Jonathon W. Juco, Jorge Reis-Filho, Joselyn Sanchez, Joseph Sparano, Joël Cucherousset, Juan Carlos Araya, Julien Adam, Justin M. Balko, Kai Saeger, Kalliopi Siziopikou, Karen Willard-Gallo, Karsten Weber, Katherine L. Pogue-Geile, Keith E. Steele, Kenneth Emancipator, Khalid AbdulJabbar, Khalid El Bairi, Kim R. M. Blenman, Kimberly H. Allison, Konstanty Korski, Lajos Pusztai, Laura Comerma, Laurence Buisseret, Lee A. D. Cooper, Leming Shi, Loes F. S. Kooreman, Luciana Molinero, M. Valeria Estrada, Magali Lacroix-Triki, Maise Al Bakir, Manu M. Sebastian, Marc van de Vijver, Marcelo Luiz Balancin, Maria Vittoria Dieci, Marie-Christine Mathieu, Marlon C. Rebelatto, Martine Piccart, Matthew G. Hanna, Matthew P. Goetz, Matthias Preusser, Mehrnoush Khojasteh, Melinda E. Sanders, Meredith M. Regan, Michael Barnes, Michael Christie, Michael Misialek, Michail Ignatiadis, Mieke van Bockstal, Miluska Castillo, Mohamed Amgad, Nadia Harbeck, Nadine Tung, Nele Laudus, Nicolas Sirtaine, Nicole Burchardi, Nils Ternes, Nina Radosevic-Robin, Oleg Gluz, Oliver Grimm, Paolo Nuciforo, Paul Jank, Paula Gonzalez-Ericsson, Pawan Kirtani, Petar Jelinic, Peter H. Watson, Peter Savas, Prudence A. Francis, Prudence A. Russell, Rajendra Singh, Rim S. Kim, Robert H. Pierce, Robert Hills, Roberto Leon-Ferre, Roland de Wind, Ruohong Shui, Sabine De Clercq, Sam Leung, Sami Tabbarah, Sandra C. Souza, Sandra O’Toole, Sandra Swain, Sarah Dudgeon, Scooter Willis, Scott Ely, Seong-Rim Kim, Shahinaz Bedri, Sheeba Irshad, Shi-Wei Liu, Shom Goel, Shona Hendry, Simonetta Bianchi, Sofia Bragança, Soonmyung Paik, Stephan Wienert, Stephen B. Fox, Stephen J. Luen, Stephen Naber, Stuart J. Schnitt, Luz F. Sua, Sunil R. Lakhani, Susan Fineberg, Teresa Soler, Thomas Gevaert, Timothy D’Alfonso, Tom John, Tomohagu Sugie, Uday Kurkure, Veerle Bossuyt, Venkata Manem, Vincente Peg Cámara, Weida Tong, Weijie Chen, Wentao Yang, William T. Tran, Yihong Wang, Yinyin Yuan, Yves Allory, Zaheed Husain, Zsuzsanna Bago-Horvath

**Affiliations:** 1 Department of Research IT, The Netherlands Cancer Institute, Amsterdam, The Netherlands; 2 Division of Tumor Biology and Immunology, The Netherlands Cancer Institute, Amsterdam, The Netherlands; 3 Division of Molecular Pathology, The Netherlands Cancer Institute, Amsterdam, The Netherlands; 4 Department of Pathology, The Netherlands Cancer Institute, Amsterdam, The Netherlands; 5 Department of Pathology, Ghent University Hospital, Ghent, Belgium; 6 Department of Biometrics, The Netherlands Cancer Institute, Amsterdam, The Netherlands; 7 Department of Medicine, Perlmutter Cancer Center, New York University School of Medicine, New York, NY USA; 8 Department of Radiation Oncology, Weill Cornell Medicine, New York, NY USA; 9 Department of Pathology and Laboratory Medicine, Weill Cornell Medicine, New York, NY USA; 10 International Breast Cancer Study Group Central Pathology Office, Department of Pathology and Laboratory Medicine, IEO European Institute of Oncology IRCCS, Milan, Italy; 11 University of Milan, Milan, Italy; 12 Department of Pathology and Laboratory Medicine, Genetic Pathology Evaluation Centre, University of British Columbia, Vancouver, BC Canada; 13 Department of Pathology and Laboratory Medicine, Indiana University Simon Cancer Center, Indianapolis, IN USA; 14 Service de Biostatistique et d’Epidémiologie, Gustave Roussy, CESP, Université-Paris Sud, Université Paris-Saclay, Villejuif, France; 15 CESP, Fac. de médecine - Univ. Paris-Sud, Fac. de médecine - UVSQ, INSERM, Université Paris-Saclay, Villejuif, France; 16 Department of Pathology, M.D. Anderson Cancer Center, Houston, TX USA; 17 Breast Cancer Translational Research Laboratory, Institut Jules Bordet, U-CRC, Université Libre de Bruxelles, Brussels, Belgium; 18 Department of Pathology, Yale School of Medicine, New Haven, CT USA; 19 Department of Medicine, Yale University School of Medicine, New Haven, CT USA; 20 Laboratory of Pathology, Center for Cancer Research, National Cancer Institute, Bethesda, MD USA; 21 Institute of Pathology, Charité Universitätsmedizin Berlin, Berlin, Germany; 22 Institute of Pathology, Philipps-University Marburg, Marburg, Germany; 23 German Breast Group, Neu-Isenburg, Germany; 24 Division of Research and Clinical Medicine, Peter MacCallum Cancer Centre, University of Melbourne, Melbourne, VIC Australia; 25 Ontario Institute for Cancer Research, Toronto, ON Canada; 26 IGMM, Edinburgh, UK; 27 Edinburgh Cancer Research Centre, Western General Hospital, Edinburgh, UK; 28 Department of Pathology and Laboratory Medicine, IRCCS Fondazion - Instituto Nazionale Tumori, Milan, Italy; 29 School of Medicine, University of Milan, Milan, Italy; 30 Department of Pathology, Brigham and Women’s Hospital, Harvard Medical School, Boston, MA USA; 31 Clinical Trials and Statistics Unit, The Institute of Cancer Research, Surrey, UK; 32 Breast Cancer Now Toby Robins Research Centre, The Institute of Cancer Research, London, UK; 33 Research and Development, Vivactiv Ltd, Chesham, Buckinghamshire UK; 34 Department of Pathology and Laboratory Medicine, University of Ottawa, Ottawa, ON Canada; 35 Department of Pathology, GZA-ZNA Ziekenhuizen, Antwerp, Belgium; 36 Department of Medical Oncology, The Netherlands Cancer Institute, Amsterdam, The Netherlands; 37 Department of Oral and Maxillofacial Diseases, Helsinki, Finland; 38 Department of Pathology, Matsuyama Shimin Hospital, Matsuyama, Japan; 39 Surgical Oncology, Baylor College of Medicine, Houston, TX USA; 40 Roche Diagnostics, Mechelen, Belgium; 41 Departments of Pathology, Genomic Medicine, Dermatology, and Translational Molecular Pathology, The University of Texas MD Anderson Cancer Center, Houston, TX USA; 42 PhenoPath Laboratories, Seattle, WA USA; 43 Research Pathology, Genentech Inc., South San Francisco, CA USA; 44 University of Turin/Candiolo Cancer Institute - FPO, IRCCS, Candiolo, Italy; 45 Case Western Reserve University, Cleveland, OH USA; 46 Louis Stokes Cleveland Veterans Health Administration Medical Center, Cleveland, OH USA; 47 Pulmonary Pathology, New York University Center for Biospecimen Research and Development, New York University, New York, NY USA; 48 Department of Pathology, Johns Hopkins Hospital, Baltimore, MD USA; 49 Department of Pathology, Istituto Europeo di Oncologia, University of Milan, Milan, Italy; 50 PathAI, Inc, Cambridge, MA USA; 51 Department of Pathology, University of Iowa Hospitals and Clinics, Iowa City, IA USA; 52 Department of Oncology, IVO, Valencia, Spain; 53 Cancer Bioinformatics Lab, Cancer Centre at Guy’s Hospital, London, UK; 54 School of Life Sciences and Medicine, King’s College London, London, UK; 55 Lund University, Skane University Hospital, Department of Clinical Sciences Lund, Oncology and Pathology, Lund, Sweden; 56 Dana Farber Cancer Institute, Boston, MA USA; 57 Institut Curie, Paris Sciences Lettres Université, Inserm U934, Department of Pathology, Paris, France; 58 Department of Surgical Pathology, Zealand University Hospital, Køge, Denmark; 59 Department of Biomedical Informatics, Emory University, GA USA; 60 Departments of Pathology and Oncology, The Johns Hopkins Hospital, Baltimore, MD USA; 61 National Surgical Adjuvant Breast and Bowel Project Operations Center/NRG Oncology, Pittsburgh, PA USA; 62 Department of Pathology, Karolinska Institutet, Karolinska, Sweden; 63 Department of Pathology, New York University Langone Medical Centre, New York, NY USA; 64 Department of Pathology and Laboratory Medicine, UNC School of Medicine, Chapel Hill, NC USA; 65 Bioinformatics and Computational Genomics Laboratory, Princess Margaret Cancer Center, Toronto, ON Canada; 66 Department of Medical Oncology, Peter MacCallum Cancer Centre, Melbourne, VIC Australia; 67 Department of Medicine, University of Melbourne, Parkville, VIC Australia; 68 Trev & Joyce Deeley Research Centre, British Columbia Cancer Agency, Victoria, BC Canada; 69 Division of Imaging, Diagnostics, and Software Reliability (DIDSR), Office of Science and Engineering Laboratories (OSEL), Center for Devices and Radiological Health (CDRH), Rockville, MD USA; 70 Department of Research, Instituto Nacional de Enfermedades Neoplásicas, Lima, Peru; 71 Department of Research, Instituto Nacional de Enfermedades Neoplásicas, Lima, 15038 Peru; 72 Providence Cancer Research Center, Portland, Oregon, USA; 73 Department of Medical Oncology, Istituto Europeo di Oncologia, Milan, Italy; 74 Roche Diagnostics, Mechelen, Belgium; 75 Department of Pathology, AZ Turnhout, Turnhout, Belgium; 76 Department of Pathology, St Vincent’s University Hospital and University College Dublin, Dublin, Ireland; 77 Department of Computational and Systems Biology, University of Pittsburgh, Pittsburgh, PA USA; 78 Cancer Research UK Lung Cancer Centre of Excellence, University College London Cancer Institute, University College London, London, UK; 79 Azienda AUSL, Regional Hospital of Aosta, Aosta, Italy; 80 Université Paris-Sud, Institut National de la Santé et de la Recherche Médicale, Villejuif, France; 81 Gustave Roussy, Universite Paris-Saclay, Villejuif, France; 82 University Hospital Halle (Saale), Institute of Pathology, Halle, (Saale) Germany; 83 Department of Pathology, UCL Cancer Institute, UCL, London, UK; 84 University College Hospitals NHS Trust, London, UK; 85 Department of Pathology, Jules Bordet Institute, Brussels, Belgium; 86 Department of Clinical Medicine, Macquarie University, Sydney, Australia; 87 HistoGeneX NV, Antwerp, Belgium and AZ Sint-Maarten Hospital, Mechelen, Belgium; 88 Oncology Clinical Development, Bristol-Myers Squibb, Princeton, NJ USA; 89 Institut für Pathologie, UK Hamburg, Germany; 90 Department of Medicine, Vanderbilt University Medical Centre, Nashville, TN USA; 91 Department of Cancer CV, Jacksonville, FL USA; 92 Department of Pathology, Memorial Sloan Kettering Cancer Center, New York, NY USA; 93 Department of Oncology, Mayo Clinic, Rochester, MN USA; 94 Roche, Tucson, AZ USA; 95 Department of Pathology, Herlev and Gentofte Hospital, Gentofte, Denmark; 96 Université Paris-Saclay, Univ. Paris-Sud, Villejuif, France; 97 Service de biostatistique et d’épidémiologie, Gustave Roussy, Villejuif, France; 98 Department of Pathology, Universidad de La Frontera, Temuco, Chile; 99 Departamento de Anatomía Patológica, Universidad de La Frontera, Temuco, Chile; 100 Tumor Pathology Department, Maria Sklodowska-Curie Memorial Cancer Center, Gliwice, Poland; 101 Pathology and Tissue Analytics, Roche, Neuherberg, Germany; 102 Department of Medical Oncology, Gustave Roussy, Villejuif, France; 103 Sunnybrook Health Sciences Centre, Toronto, ON Canada; 104 Tehran University of Medical Sciences, Tehran, Iran; 105 Pathology Department, Instituto de Investigación Sanitaria Fundación Jiménez Díaz (IIS-FJD), Madrid, Spain; 106 GEICAM-Spanish Breast Cancer Research Group, Madrid, Spain; 107 Translational Research, Montreal, Canada; 108 Computational Pathology Group, Department of Pathology, Radboud University Medical Center Nijmegen, The Netherlands; 109 Oncology Biomarker Development, Genentech-Roche, Neuherberg, Germany; 110 Division of Medical Oncology, Department of Medicine, University of Colorado Anschutz Medical Campus, Aurora, CO USA; 111 Centre de Lutte Contre le cancer - Centre Jean Perrin, Clermont-Ferrand, France; 112 Department of Pathology, Hospital de Oncología Maria Curie, Buenos Aires, Argentina; 113 Directorate of Surgical Pathology, SA Pathology, Adelaide, Australia; 114 Department of Pathology, GZA-ZNA Ziekenhuizen, Wilrijk, Belgium; 115 University of Milano, Instituto Europeo di Oncologia, IRCCS, Milano, Italy; 116 Division of Early Drug Development for Innovative Therapy, IEO, European Institute of Oncology IRCCS, Milan, Italy; 117 Department of Imaging and Pathology, Laboratory of TranslCal Cell & Tissue Research, Leuven, Belgium; 118 KU Leuven- University Hospitals Leuven, Department of Pathology, Leuven, Belgium; 119 Department of Pathology, University Hospital of Antwerp, Antwerp, Belgium; 120 Paula I, Breast Cancer Research Program, Vanderbilt University Medical Center, Nashville, TN USA; 121 Translational Health Sciences, Department of Cellular Pathology, North Bristol NHS Trust, University of Bristol, Bristol, UK; 122 Medical Oncology, Department of Medicine, Cedars-Sinai Medical Center, Los Angeles, CA USA; 123 Helsinki University Central Hospital, Helsinki, Finland; 124 Department of Clinical Pathology, Akademiska University Hospital, Uppsala, Sweden; 125 Department of Pathology, National Taiwan University Hospital, Taipei, Taiwan; 126 Department of Oncology, National Taiwan University Cancer Center, Taipei, Taiwan; 127 Graduate Institute of Oncology, College of Medicine, National Taiwan University, Taipei, Taiwan; 128 International Agency for Research on Cancer (IARC), World Health Organization, Lyon, France; 129 Department of Pathology, Sanatorio Mater Dei, Buenos Aires, Argentina; 130 Institute of Pathology, Medical University of Graz, Graz, Austria; 131 Department of Oncology, National Cancer Centre Singapore, Singapore, Singapore; 132 Department of Pathology, Brigham and Women’s Hospital, Boston, MA USA; 133 Department of Pathology, Netherlands Cancer Institute, Amsterdam, The Netherlands; 134 PathAI Inc, Cambridge, MA USA; 135 Visiopharm A/S, Hørsholm, Denmark; 136 DTU Compute, Department of Applied Mathematics, Technical University of Denmark, Lyngby, Denmark; 137 Nuffield Department of Population Health, University of Oxford, Oxford, UK; 138 Department of Medical Oncology, University Hospitals Bristol NHS Foundation Trust, Bristol, UK; 139 R&D UNICANCER, Paris, France; 140 Translational Sciences, MedImmune, Gaithersberg, MD USA; 141 Breast Unit, Champalimaud Clinical Centre, Lisboa, Portugal; 142 Department of Pathology, Massachusetts General Hospital, Boston, MA USA; 143 Department of Laboratory Medicine and Pathology, Mayo Clinic, Rochester, MN USA; 144 Biomedical Informatics Department, Stony Brook University, Stony Brook, NY USA; 145 Department of Oncology and Pathology, Karolinska Institutet and University Hospital, Solna, Sweden; 146 Department of Medicine, Clinical Division of Oncology, Comprehensive Cancer Centre Vienna, Medical University of Vienna, Vienna, Austria; 147 Leicester Cancer Research Centre, University of Leicester, Leicester, and MRC Toxicology Unit, University of Cambridge, Cambridge, UK; 148 Merck & Co., Inc, Kenilworth, USA; 149 Human Oncology and Pathogenesis Program, Memorial Sloan Kettering Cancer Center, New York, NY USA; 150 Department of Research, Instituto Nacional de Enfermedades Neoplasicas, Lima, 15038 Peru; 151 Department of Medicine, Department of Obstetrics and Gynecology and Women’s Health, Albert Einstein Medical Center, Bronx, USA; 152 GHI Le Raincy-Montfermeil, Chelles, Île-de-France, France; 153 Department of Pathology, Gustave Roussy, Grand Paris, France; 154 Departments of Medicine and Cancer Biology, Vanderbilt University Medical Centre, Nashville, TN USA; 155 VMscope GmbH, Berlin, Germany; 156 Department of Pathology, Breast Pathology Section, Northwestern University, Chicago, IL USA; 157 Molecular Immunology Unit, Institut Jules Bordet, Université Libre de Bruxelles, Brussels, Belgium; 158 NSABP/NRG Oncology, Pittsburgh, PA USA; 159 Division of Molecular Pathology, Centre for Evolution and Cancer, The Institute of Cancer Research, London, UK; 160 Cancer Biomarkers Working Group, Faculty of Medicine and Pharmacy, Université Mohamed Premier, Oujda, Morocco; 161 Yale Cancer Center Genetics, Genomics and Epigenetics Program, Yale School of Medicine, New Haven, CT USA; 162 Pathology Department, Stanford University Medical Centre, Stanford, CA USA; 163 Pathology and Tissue Analytics, Roche Innovation Centre Munich, Penzberg, Germany; 164 Pathology Department, Hospital del Mar, Parc de Salut Mar, Barcelona, Spain; 165 Department of Pathology, Northwestern University Feinberg School of Medicine, Chicago, IL USA; 166 Center for Pharmacogenomics and Fudan-Zhangjiang, Center for Clinical Genomics School of Life Sciences and Shanghai Cancer Center, Fudan University, Fudan, China; 167 GROW - School for Oncology and Developmental Biology, Maastricht University Medical Centre and Department of Pathology, Maastricht University Medical Centre, Maastricht, The Netherlands; 168 Biorepository and Tissue Technology Shared Resources, University of California San Diego, San Diego, CA USA; 169 Department of Pathology, Gustave Roussy, Villejuif, France; 170 Departments of Epigenetics and Molecular Carcinogenesis, The University of Texas MD Anderson Cancer Center, Houston, TX USA; 171 Department of Pathology, Academic Medical Center, Amsterdam, The Netherlands; 172 Department of Pathology, University of São Paulo, São Paulo, Brazil; 173 Hospital das Clínicas, Sao Paulo, Brasil; 174 Department of Surgery, Oncology and Gastroenterology, University of Padova, Padua, Italy; 175 Department of Medical Biology and Pathology, Gustave Roussy Cancer Campus, Villejuif, France; 176 Institut Jules Bordet, Universite Libre de Bruxelles, Brussels, Belgium; 177 Roche Tissue Diagnostics, Digital Pathology, Santa Clara, CA USA; 178 Department of Pathology, Microbiology and Immunology, Vanderbilt University Medical Centre, Nashville, TN USA; 179 Division of Biostatistics, Dana-Farber Cancer Institute, Boston, MA USA; 180 Harvard Medical School, Boston, MA USA; 181 Roche Diagnostics Information Solutions, Belmont, CA USA; 182 Department of Anatomical Pathology, Royal Melbourne Hospital, Parkville, VIC Australia; 183 Vernon Cancer Center, Newton-Wellesley Hospital, Newton, MA USA; 184 Department of Medical Oncology, Institut Jules Bordet, Université Libre de Bruxelles, Brussels, Belgium; 185 Service de pathologique, Cliniques universitaires Saint-Luc, Bruxelles, Belgique; 186 Department of Biomedical Informatics, Emory University School of Medicine, Atlanta, GA USA; 187 Breast Center, Dept. OB&GYN and CCC (LMU), University of Munich, Munich, Germany; 188 Division of Hematology-Oncology, Beth Israel Deaconess Medical Center, Boston, MA USA; 189 University of Leuven, Leuven, Belgium; 190 Department of Pathology, Institut Jules Bordet, Université Libre de Bruxelles, Brussels, Belgium; 191 German Breast Group, Neu-Isenburg, Germany; 192 Department of Surgical Pathology and Biopathology, Jean Perrin Comprehensive Cancer Centre, Clermont-Ferrand, France; 193 Johanniter GmbH - Evangelisches Krankenhaus Bethesda Mönchengladbach, West German Study Group, Mönchengladbach, Germany; 194 Molecular Oncology Group, Vall d’Hebron Institute of Oncology, Barcelona, Spain; 195 Department of Pathology, University of Marburg, Marburg, Germany; 196 Breast Cancer Program, Vanderbilt-Ingram Cancer Center, Vanderbilt University Medical Center, Nashville, TN USA; 197 Department of Histopathology, Manipal Hospitals Dwarka, New Delhi, India; 198 Department of Pathology and Laboratory Medicine, University of British Columbia, Vancouver, BC Canada; 199 The Sir Peter MacCallum Department of Oncology, University of Melbourne, Melbourne, VIC Australia; 200 Peter MacCallum Cancer Centre, Melbourne, VIC Australia; 201 Sir Peter MacCallum Department of Oncology, University of Melbourne, Melbourne, VIC Australia; 202 Department of Anatomical Pathology, St Vincent’s Hospital Melbourne, Fitzroy, VIC Australia; 203 Icahn School of Medicine at Mt. Sinai, New York, NY USA; 204 NRG Oncology/NSABP, Pittsburgh, PA USA; 205 Cancer Immunotherapy Trials Network, Central Laboratory and Program in Immunology, Fred Hutchinson Cancer Research Center, Seattle, WA USA; 206 Clinical Trial Service Unit & Epidemiological Studies Unit, University of Oxford, Oxford, UK; 207 Department of Pathology, Fudan University Cancer Center, Shanghai, China; 208 Department of Pathology, GZA-ZNA Hospitals, Antwerp, Belgium; 209 University of British Columbia, Vancouver, BC Canada; 210 Department of Radiation Oncology, Odette Cancer Centre, Sunnybrook Research Institute, Toronto, ON Canada; 211 Merck Oncology, Kenilworth, NJ USA; 212 The Cancer Research Program, Garvan Institute of Medical Research, Darlinghurst, NSW Australia; 213 Georgetown University Medical Center, Washington, DC USA; 214 FDA/CDRH/OSEL/Division of Imaging, Diagnostics, and Software Reliability, Silver Spring, MD USA; 215 Department of Molecular and Experimental Medicine, Avera Cancer Institute, Sioux Falls, SD USA; 216 Translational Medicine, Bristol-Myers Squibb, Princeton, NJ USA; 217 National Surgical Adjuvant Breast and Bowel Project Operations Center/NRG Oncology, Pittsburgh, PA USA; 218 Anatomic Pathology, Boston, MA USA; 219 Guy’s Hospital, London, UK; 220 King’s College London, London, UK; 221 Peking University First Hospital Breast Disease Center, Beijing, China; 222 Department of Pathology, Peter MacCallum Cancer Centre, Melbourne, VIC Australia; 223 Dipartimento di Scienze della Salute (DSS), Firenze, Italy; 224 Department of Oncology, Champalimaud Clinical Centre, Lisbon, Portugal; 225 Charité - Universitätsmedizin Berlin, corporate member of Freie Universität Berlin, Humboldt-Universität zu Berlin, and Berlin Institute of Health, Institute of Pathology, Berlin, Germany; 226 Department of Pathology and Laboratory Medicine, Tufts Medical Center, Boston, MA USA; 227 Dana-Farber Cancer Institute, Boston, MA USA; 228 Department of Pathology, Fundación Valle del Lili, Cali, Valle del Cauca, Colombia; 229 The University of Queensland Centre for Clinical Research and Pathology Queensland, Brisbane, QLD Australia; 230 Department of Pathology, Montefiore Medical Center and the Albert Einstein College of Medicine, Bronx, NY USA; 231 Department of Pathology, University Hospital of Bellvitge, Oncobell, IDIBELL, L’Hospitalet del Llobregat, Barcelona, 08908 Catalonia Spain; 232 Department of Development and Regeneration, Laboratory of Experimental Urology, KU Leuven, Leuven, Belgium; 233 Department of Pathology, Memorial Sloan Kettering Cancer Center, New York, NY USA; 234 Department of Medical Oncology, Austin Health, Heidelberg, VIC Australia; 235 Department of Surgery, Kansai Medical School, Hirakata, Japan; 236 Roche Tissue Diagnostics, Digital Pathology, Santa Clara, CA USA; 237 Department of Pathology, Massachusetts General Hospital, Boston, MA USA; 238 Pathology Department, H.U. Vall d’Hebron, Barcelona, Spain; 239 Division of Bioinformatics and Biostatistics, US Food and Drug Administration, Silver Spring, MD USA; 240 Department of Pathology and Laboratory Medicine, Rhode Island Hospital and Lifespan Medical Center, Providence, RI USA; 241 Université Paris-Est, Créteil, France; 242 Praava Health, Dhaka, Bangladesh; 243 Department of Pathology, Medical University of Vienna, Vienna, Austria

**Keywords:** Breast cancer, Biomarkers, Tumour biomarkers, Tumour immunology

## Abstract

Stromal tumor-infiltrating lymphocytes (sTILs) are a potential predictive biomarker for immunotherapy response in metastatic triple-negative breast cancer (TNBC). To incorporate sTILs into clinical trials and diagnostics, reliable assessment is essential. In this review, we propose a new concept, namely the implementation of a risk-management framework that enables the use of sTILs as a stratification factor in clinical trials. We present the design of a biomarker risk-mitigation workflow that can be applied to any biomarker incorporation in clinical trials. We demonstrate the implementation of this concept using sTILs as an integral biomarker in a single-center phase II immunotherapy trial for metastatic TNBC (TONIC trial, NCT02499367), using this workflow to mitigate risks of suboptimal inclusion of sTILs in this specific trial. In this review, we demonstrate that a web-based scoring platform can mitigate potential risk factors when including sTILs in clinical trials, and we argue that this framework can be applied for any future biomarker-driven clinical trial setting.

## Introduction

Clinical trials in cancer research are increasingly incorporating biomarkers, for example, as an inclusion criterion or for stratification of patients to control for confounding factors. Practical challenges, such as interobserver variation in the assessment of biomarkers *during* the execution of the trial, are often overlooked. If not handled appropriately, these challenges can limit the effectiveness and ability to complete the biomarker and drug development process. According to Hall et al.^1^, the risks inherent to biomarker integration can be divided into risks to patients, operational risks, and direct risks to biomarker development. A practical risk-management framework developed by a National Cancer Institute (NCI), National Cancer Research Institute (NCRI), and European Organization for Research and Treatment of Cancer (EORTC) Working Group^1^ was proposed to manage the risks inherent to biomarker integration into clinical trials.

Stromal tumor-infiltrating lymphocytes (sTILs) have been strongly associated with prognosis in early-stage triple-negative breast cancer (TNBC) and HER2-positive breast cancer. In addition, sTILs are predictive for neo-adjuvant chemotherapy response in early breast cancer^2,3^. Furthermore, sTILs correlate with outcome after immune checkpoint blockade in metastatic TNBC^4–6^. The readout of sTILs, however, can be challenging impeding its effective use as a biomarker and its usage in the clinic^7^. The International Immuno-Oncology Biomarker Working Group (hereafter called the TIL Working Group) has provided guidelines for the scoring of sTILs in breast cancer^8^, and the St. Gallen Breast Cancer Conference of 2019 endorsed sTILs being routinely characterized in TNBC and reported according to these guidelines^8^.

## Risks associated with integration of biomarkers in clinical trials

In contemporary clinical research there is an increasing trend toward the use of biomarker results obtained in daily practice to select patients for inclusion in clinical trials. Although biomarker research is more and more prominent in clinical trials, most biomarkers will not make into the clinic^9^. Therefore, continuous monitoring of the predefined risks and the solutions can improve the quality of the biomarker, which can be applied in a clinical trial setting, as well as in daily practice. The recommendations of the TIL Working Group^8,10^ for appropriate scoring, and the risk-management framework of the NCI, NCRI, and EORTC Working Groups^1^ will help to effectively and efficiently improve the incorporation of biomarkers in clinical trials in first instance.

Several risks are associated with biomarker development and integration of biomarkers in clinical trials. Roughly, risks can be divided into three categories: risks to patient safety, operational risks, and risks to biomarker development. Not all risks are applicable to all clinical trials and upon designing a biomarker-incorporating clinical trial, risks should be defined and mitigation approaches formulated. It is highly recommended that during a clinical trial, risks are not only pre-identified but are also continuously monitored to prevent stagnation in biomarker development^1^. For example, incorporating biomarkers in a large multi-center international clinical trial involves different risks than a small single-center trial. In the first case, there might be different legislation regarding data confidentiality, and inter-laboratory variability can be an issue. When incorporating a biomarker as inclusion criterion or stratification factor in clinical trials, rapid turnaround times are needed and the highest level of quality is necessary for correct interpretation of the results. In the next steps of biomarker development, high-quality results are needed to ensure implementation in daily clinical practice.

## Use of digital pathology in clinical trials and development of a novel web application

In larger trials, usually phase II–III, central pathology review (CPR) plays an important role in the reliable assessment of biomarker scoring. However, logistical issues, such as the sending of tumor blocks or slides, can be time consuming, costly for the pathology laboratory, and error prone with significant consequences for patient inclusion if the wrong material is sent to the central lab. Digital sharing of histology slides and patient data simplifies logistics for CPR^11^. Besides digital sharing and scoring of slides, digital image analysis and machine learning approaches are emerging in clinical research^12,13^. The use of digital pathology or digital evaluation of histology slides most prominently mitigates risks associated with operational processes. It can reduce the number of missing samples, since the sharing of material is simplified; it enables rapid turnaround times; reduces manual errors; and can streamline local versus central assessment of biomarker.

For clinicians and researchers to use digital pathology, applications and websites should be user-friendly and intuitive. As an example, a web-based tool called Slide Score (www.slidescore.com) was developed as a cross-platform web application to facilitate the scoring of whole slide images and tissue microarray (TMA) cores. Application programming interface (API) was implemented that allowed programmatic administration of studies, uploading slides, fetching results, and retrieving pixel data for regions of images. This API enabled automating creation of new studies from internal database system for managing biobanking workflows. Additionally, a plugin was developed for QuPath^14^—open-source image analysis software—which uses this API to run image analysis algorithms on slides stored on the Slide Score platform avoiding the need to download the slides. This web-based platform was used in high-impact projects^6,15^, for example, for the digital scoring of biomarkers in the first stage of the TONIC trial^6^, and the estimation of the immune infiltrate of tumors of melanoma patients used for single-cell sequencing^15^. Furthermore, the web-based platform is currently used for several other types of research, such as interrater variability studies, retrospective TMA, and whole slide scoring and prospective biomarker scoring.

## Design of a workflow to mitigate risks associated with biomarker development: an example

**Fig. 1:**
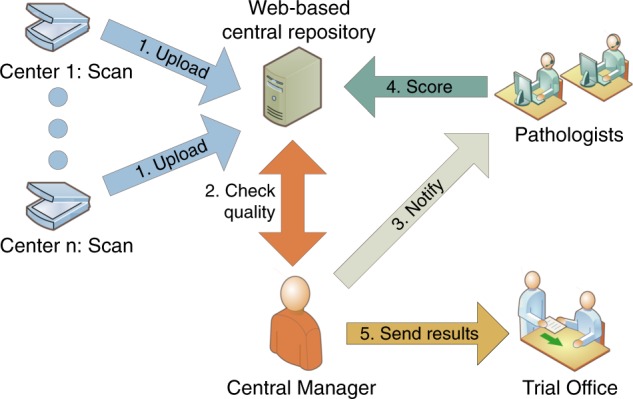
Organization of a workflow for reliable and timely biomarker scoring in a general single-center or multi-center trial. Personnel at individual centers scan the slides after processing by the local pathology department. Digital slides are uploaded to a central web-based repository, such as Slide Score. A study-specific identifier is assigned to each sample. The central manager is notified by the system when new slides are available and requests pathologists to review it. When a consensus score is obtained, the trial office is notified for randomization of the patient.

We identified seven distinct risks with the risk-management framework published by Hall et al.^1^ as possibly interfering with the quality and integration of prospective sTILs scores in a clinical trial, and designed our workflow accordingly (Table 1). These risks are specific for this trial, but some of them are applicable also to other trials. They span all three categories mentioned above^1^ and included (1) poor-quality biopsies, (2) possible loss of data confidentiality, (3) interrater variability, (4) poor sample quality, (5) poor scoring quality, (6) delay in patient registration, and (7) manual errors (Table 1). We then defined solutions to mitigate these risks and integrated these solutions in a workflow that can be applied across clinical trials and across biomarkers (Fig. 1). The workflow can be modified according to local guidelines, research questions, and clinical trial designs. We used the following workflow to obtain timely and reliable sTILs scores (summary in Supplementary Fig. 1).

**Table 1:** Risks with possible high impact identified in a phase II immunotherapy trial^6^ based on the perspectives of Hall et al.^1^ with our approach to mitigation of that risk.

	Type of risk	Risk	Description of risk	Mitigation approach
1.	Risks to patients	No stroma or tumor cells in biopsy	Discomfort and risks associated with a sampling intervention	Take multiple biopsies from one lesion at the same time (a minimum of three biopsies per lesion^9^) and check amount of tumor cells before analysis of sTILs and inclusion in the trial
2.	Risks to patients	Loss of data confidentiality	Patient samples sent to multiple institutions and reviewers	Pseudonymization should be applied, hide slide labels, implement strict access control, ensure no metadata is linked to a slide
3.	Risks to biomarker development	Inter-laboratory variability and interobserver variability	Different methodologies used to score slides, interrater variability	Use of international guidelines for scoring and training, use consensus score of four expert pathologists from three institutes
4.	Operational risks	Failure of sample collection, processing, and quality	Missing or poor-quality samples resulting in poor consensus scoring	Standardized tissue processing, workflow management
5.	Operational risks	Inadequate image quality or no ability to access image	Missing, poor, or inaccurate scoring	Track the scores of all pathologists and notify when scores are inconsistent
6.	Operational risks/risks to patients	Long turnaround time	Delay in patient randomization and treatment	Timeline tracking incorporated in workflow
7.	Operational risks	Data management failure	Errors in collecting manual scores, typos, data conversion issues	Structured digital scores from pathologist to the analyst

After obtaining informed consent of a patient, three biopsies of one metastatic lesion (lymph node, skin, liver, or other) were obtained in this trial. Previous research has shown that three 14 G core needle biopsies should be sufficient for accurate breast cancer diagnosis^16^. A hematoxylin and eosin (H&E)-stained slide of one biopsy was then evaluated, to ensure that the biopsy contained enough tumor cells (more than 100 cells) for further analysis (risk 1). Next, a high-resolution digital scan was obtained and automatically pseudonymized with study-specific identifiers (risk 2) before uploading to Slide Score. Display of the original labels was masked to ensure confidentiality of all data within Slide Score (Supplementary Fig. 2b). Pathologists and administrators had to login with their username and password to access the slides and were able to add a two-factor authentication application. Four well-trained breast pathologists, based in three different institutes and in two different countries, were notified via email to score each slide using existing sTIL scoring guidelines of the TIL Working Group^8,10^ to reduce interrater variability (risk 3). sTILs are scored as the percentage of lymphocytes in the total stromal area (in close proximity of the tumor cells). Interrater variability can lead to bias in the results, when assessment of a biomarker is skewed towards either the lower or higher ranges. When there was a disagreement (using a 5% cut-off) a concordance-score was agreed upon (Supplementary Fig. 1). Low-quality, inaccurate collection or processing of samples can result in low sample availability and introduce batch effects or bias in the results (risk 4) and lead to non-consistent scores (risk 5). High quality of samples was ensured by standardization of our workflow in which all steps were performed in the same manner for every biopsy (Supplementary Fig. 1). Oversight of the entire workflow by one person, referred to as the central manager, is essential for timely identification of technical errors. The central manager tracked the timing of the biopsies, notified the pathologists immediately after the scan was uploaded and sent reminders if necessary, kept track of the scores and timing, and noted the score in the patient record for trial office notification. We predefined acceptable timeframes for obtaining the scores of the reviewers and tracked these during the study progress (risk 6; Supplementary Fig. 1). Pathologists were notified via email the next working day when the slide was not scored yet to minimize the waiting period to start treatment (risk 6). Finally, using Slide Score, we reduced the risks of typos and other manual errors by collecting all slides within one online study group (collection of slides) and a customized scoring form was built to standardize scores and obtain structured data (risk 7).

## Implementation of workflow in the TONIC trial

The TONIC trial (NCT02499367)^6^ is a phase II, non-comparative randomized multi-cohort single-center trial (full title: Adaptive phase II randomized non-comparative Trial Of Nivolumab after Induction treatment in TNBC patients), designed to assess the efficacy of induction of an anti-cancer immune response by low-dose chemotherapy or irradiation to increase response to anti-PD-1 in patients with metastatic TNBC. In the first part of the trial^6^, patients with metastatic TNBC were randomized to nivolumab (1) without induction or two-week low-dose induction, with (2) irradiation (3 × 8 Gy), (3) cyclophosphamide, (4) cisplatin, or (5) doxorubicin, all followed by nivolumab (anti-PD-1; 3 mg/kg). Based on a Simon’s two-stage design^17^ and prespecified pick-the-winner criteria, only the doxorubicin cohort was allowed to continue in the second part of the trial^6^. In the second part of the TONIC trial, patients were randomized between anti-PD-1 monotherapy (control group) and two cycles of low-dose doxorubicin (15 mg flat dose, weekly), followed by anti-PD-1 (Supplementary Fig. 2a). Randomization was stratified for sTILs. Stratification is done by dividing patients in two categories, namely sTIL_high_ (equal or exceeding 5%) and sTIL_low_ (lower than 5%). The cut-off was determined based on data obtained in the first part of the TONIC trial, in which we observed that sTILs were predictive of response to anti-PD-1, both continuous and when a cut-off of 5% was used^6^. These data confirmed the predictive value of sTILs of at least 5% in another trial, which tested the efficacy of anti-PD-1 in patients with metastatic TNBC^4^. The full protocol, including four amendments, and the informed consent form were approved by the medical-ethical committee of The Netherlands Cancer Institute. All patients provided written informed consent before enrollment. The trial was registered on 17 August 2015. The 47 patients of the second part of the trial were randomized between March 2018 and July 2019. Full eligibility criteria and trial procedures have been described previously^6^.

In the second part of the TONIC trial, we could implement our workflow with a focus on accurate and reproducible sTIL scores within a reasonable timeframe after a biopsy was taken (72 h). For all 47 patients included in the trial, reliable sTIL scores were obtained with 45 biopsies scored within the 72-h timeframe (Supplementary Fig. 3). During the course of the study, the server of Slide Score was available 99.9% of the time. Five biopsies had to be re-evaluated due to a discrepancy in the categorical scores, when not all pathologists agreed on the appropriate category of the sTIL score (lower than 5% versus higher or equal to 5%). In three of these cases the score of one pathologist was higher (5 or 10%) than the score of the other two or three pathologists (0–3%). The average sTIL score was obtained and the pathologist causing the disagreement was notified. In the fourth and fifth case, two pathologists scored 5 and 10%, whereas the other pathologists scored 1%. All four pathologists were notified of the disagreement and a consensus score of 5% was obtained. We observed an intraclass correlation coefficient of 0.94 (95% confidence interval (CI): 0.91–0.97) for sTILs as a continuous variable. Interrater agreement for the categorical variable used in the stratification (sTILs <5% or ≥5%) was 0.86 (multirater Fleiss’ *κ*^18^; 95% CI: 0.73–1; Supplementary Fig. 2c). In the anti-PD-1 monotherapy cohort, we observed that 13 out of 23 patients (56.5 %) had sTILs below 5%, as compared to 15 out of 24 patients in the doxorubicin cohort (62.5 %; Fisher’s exact test *p* value 0.77). The distribution of the sTIL scores is depicted in Supplementary Fig. 2d. These data indicate effective stratification based on the cut-off of 5%, but a slightly uneven distribution in the higher ranges of sTIL scores (10% or higher) inherent to the use of our cut-off. We observed a median time from biopsy until the scanning of the H&E slide of 30 h (range 24–98 h) and a median time from the biopsy until at least three scores were obtained of 43 h (range 27–106 h). In total, the median time from biopsy until registration in the patient records was 49 h (range 41–106 h; Supplementary Fig. 2e), with 96% of biopsies scored within 72 h. Two biopsies were not scored within the 72-h time limit, due to additional processing of one sample and one delay in registration time due to the absence of the central manager (Supplementary Figs 1 and 3).

## Advantages and limitations of a web-based risk-mitigation workflow

Our proposed solutions involved standardization of our workflow, obtaining digital images and the use of a web-based tool such as Slide Score for the managing and scoring of digital images. Anticipating the incorporation of digital images in routine diagnostics, our workflow shows that it is feasible for a pathologist to score digital images with high reliability. Moreover, a web-based tool can facilitate the process of coordinated uploading of digital images, pseudonymizing slides, and regulate access to studies and proper data management. Web-based platforms are therefore of high interest in biomarker research and can help with automation that can be transferred to clinical practice in the future.

In this study, we obtained sTIL scores within 72 h after a biopsy was taken, which is a reasonable timeframe for clinicians to start randomization of patients to treatment arms in a clinical trial. We observed an excellent interrater agreement score between our panel of four expert pathologists. In an accompanying paper^7^ we demonstrate using data from three RING studies of the TIL Working Group that the concordance achieved using a risk-management approach as detailed in this study is substantially higher than observed outside this risk-management perspective as observed in the three RING studies and in other published studies^19,20^. However, our sample size is small and the four pathologists in the current study were trained and experienced in the scoring of sTILs in breast cancer. Also, the biopsies used in this study were checked for containing sufficient tumor cells (≥100 cells) before the slide was scored for sTILs, which could have further improved our results. In the future, it is to be expected that computational workflows will further improve the scoring of sTILs^13^. Although we obtained reliable and timely results in 96% of cases, the presence of a central manager is crucial. In one case there was a delay in registration time due to the absence of the central manager. The manual intervention of quality checks, processing of the slides, and data cannot be circumvented in our workflow.

Stratification in this study was performed using sTILs as a binary variable (lower than 5% versus higher or equal to 5%). Consequently, we observed an uneven distribution in continuous sTILs scores between the cohorts (Supplementary Fig. 2d). This was mainly due to more patients with sTILs scores above 10% in the anti-PD-1 monotherapy cohort. Inherent to the use of a binary cut-off for stratification, the median of the continuous measurement might still differ between cohorts. Alternatively, multiple categories for the same variable can be used in stratification. However, this approach generates more strata, with lower number of patients in each stratum, possibly leading to an imbalance in distribution^21,22^. Moreover, at the time of writing of this paper no cut-offs for sTILs are established and/or properly validated for predictive purposes.

During the trial, we continuously monitored whether our strategy was still feasible within the set timeframe by means of regular evaluation by the pathologists and the study coordinators. This led to rapid adjustment of the workflow if needed, ensuring the quality of the sTIL scores. For example, pathologists could easily login remotely and score a digital H&E outside the hospital ensuring that sTILs were still scored within 72 h after biopsy. Ongoing evaluation during the clinical trial is of critical importance for risk mitigation in biomarker research^1^.

## Future applications of the workflow

Our strategy can serve as a template for risk management and mitigation of all identified risks in future clinical trials incorporating biomarkers for inclusion, enrichment, or stratification. By no means will risks identified in this study be similar for all clinical trials. Each trial will have its own risks that need to be mitigated, although there will be similarities between the risks across clinical trials. Defining the risks that come with biomarker development will help tested biomarkers eventually make their way to the clinic. However, one may even argue that a similar risk-management strategy can be applied in daily practice. In the BELLINI trial (NCT03815890), two cycles of neo-adjuvant anti-PD-1 are administered in patients with early-stage TNBC or luminal B breast cancer. All patients are required to have at least 5% sTILs in the pretreatment biopsy and patients are thereafter stratified in three sTIL categories. Our workflow will be used to ensure timely and reliable sTIL scores for the right patient selection. By using our workflow, scoring of sTILs is highly standardized, allowing also smaller centers with less extensive experience in sTILs scoring to participate in a clinical trial.

## Conclusions

In contemporary clinical research there is an increasing trend toward the use of biomarker results obtained in daily practice to select patients for inclusion in clinical trials. Therefore, continuous monitoring of the predefined risks and the solutions can improve the quality of the biomarker, as can be applied in a clinical trial setting, as well as in daily practice. The recommendations of the TIL Working Group^8,10^ for appropriate scoring, the risk-management framework of the NCI, NCRI, and EORTC Working Groups^1^, as well as our proposed strategies to reduce risks will help to effectively and efficiently improve the incorporation of biomarkers in clinical trials in first instance, herewith illustrated using sTILs as a paradigm of this development.

## Supplementary information

Supplementary Material

## Data Availability

The data that support the findings of this study are available from the corresponding author upon reasonable request.
