# Supplementary material for: Application of a risk-management framework for integration of stromal tumor-infiltrating lymphocytes in clinical trials
Source: NPJ Breast Cancer. 2020 May 12;6:15. doi: 10.1038/s41523-020-0155-1 (PMC7217941; doi:10.1038/s41523-020-0155-1)
Supplement: Supplementary file 1 — Supplementary Material [file 41523_2020_155_MOESM1_ESM.pdf]

Supplementary Material

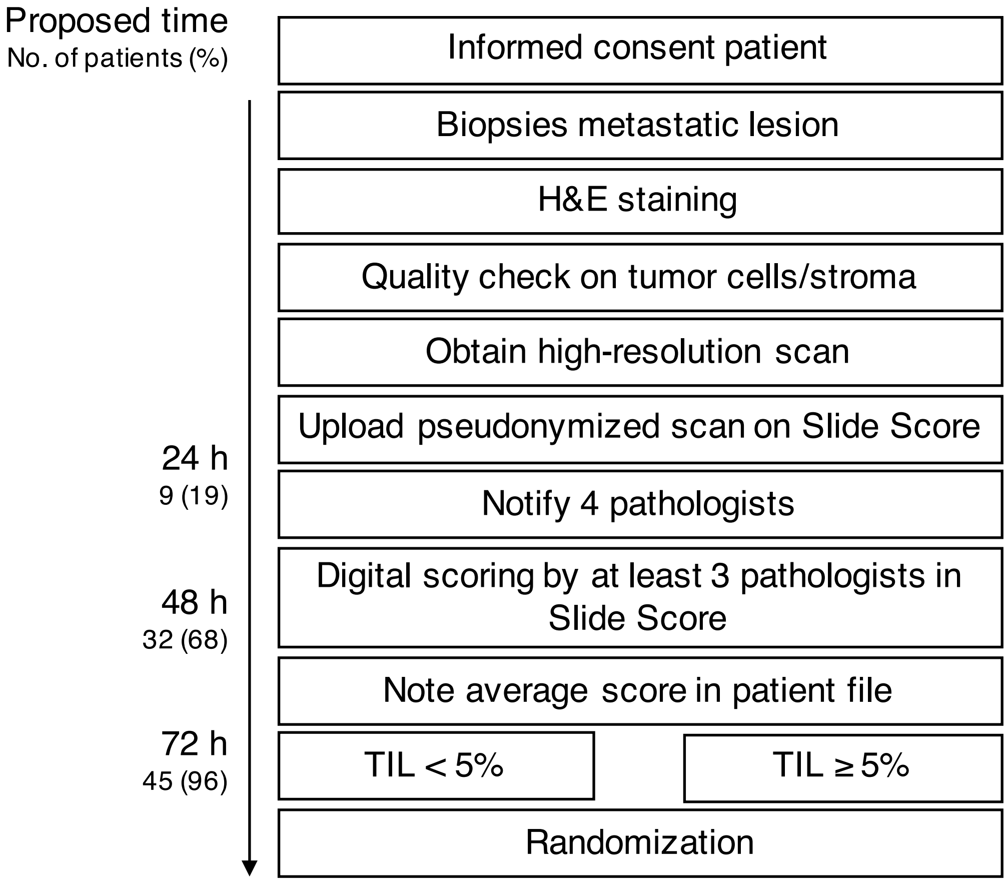

Supplementary Figure 1: Workflow for risk mitigation for the use of sTILs as a stratification factor in the TONIC-trial. Every block represents one step in the workflow. On the left side of the workflow, the proposed timeline is depicted. Numbers below the timestamps reflect the number of patients and percentage of total patients that finalized the step within the proposed time.

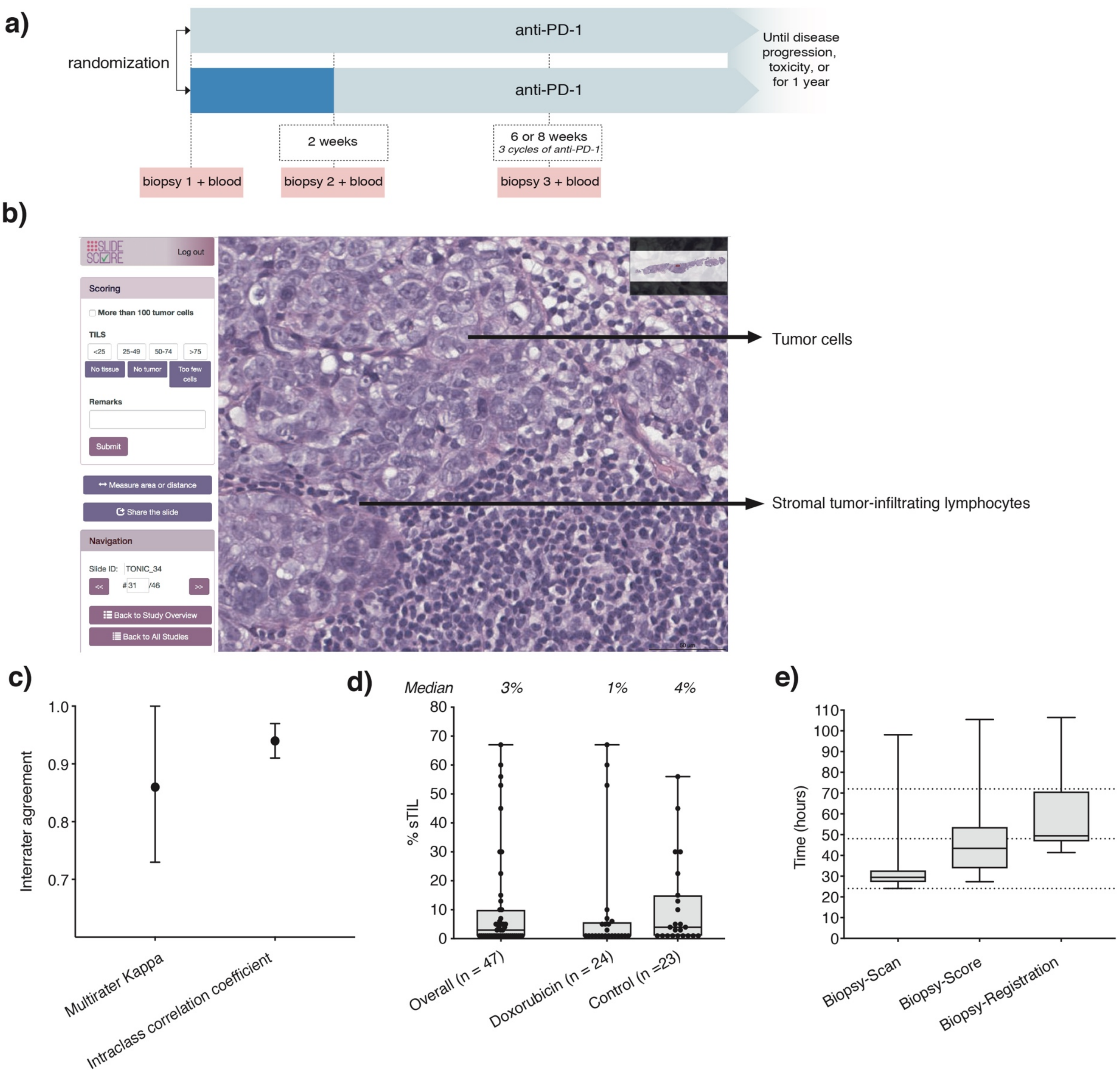

Supplementary Figure 2: Implementation of the workflow in the second part of the TONIC-trial. a) Setup of part 2 of the adaptive phase II TONIC-trial[5]. Patients are randomized between anti-PD-1 monotherapy (control cohort) or start with two cycles of low-dose doxorubicin followed by anti-PD-1. Before the start of treatment biopsies are taken. **b) User interface of Slide Score.** A customized sTILs scoring form is depicted in the image. Users can zoom in on the whole image with high resolution. A representative lymph node biopsy is displayed. **c) Interrater agreement**

sTILs. The intraclass correlation coefficient (ICC) is calculated over continuous sTILs of all raters. Fleiss' Kappa is calculated over categorical sTILs (5% or higher versus lower than 5%) of all raters. Error bars represent the 95% confidence intervals. **d) Distribution of sTILs over all patients and per cohort.** The final sTILs scores that are used for stratification are depicted (average of three or four pathologists). Every dot represents one score, boxplots represent the median with 25-75% percentile, error bars represent the range. **e) Turnaround time sTILs.** Depicted is the time in hours that it takes from the biopsy to the scan, the biopsy to the final score of the panel of pathologists and the biopsy to the registration of sTILs in the patient records (availability of scores). Boxplots represent the median with 25-75% percentile, error bars represent the range.

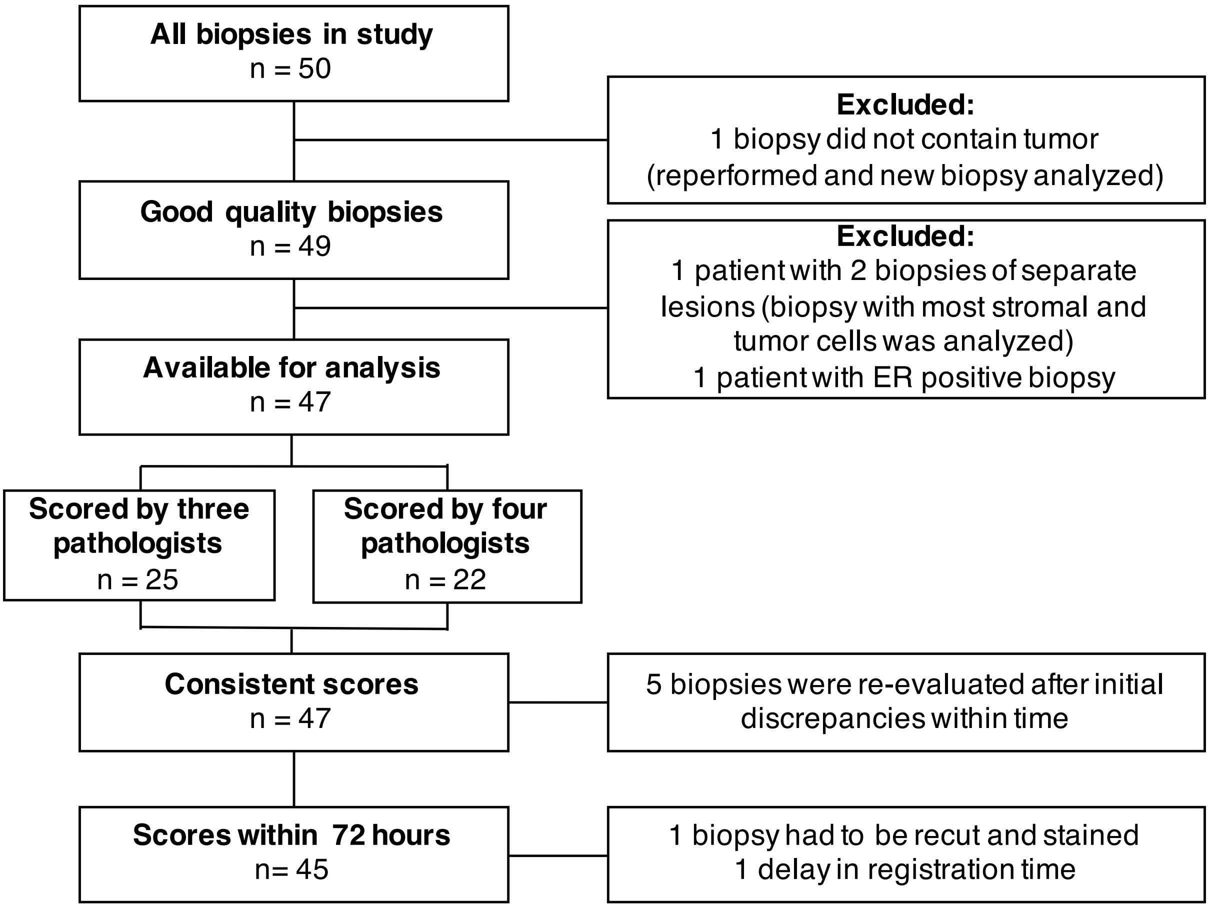

Supplementary Figure 3: Diagram of biopsies and sTILs scores available for stratification in the TONIC-trial.
